# Restricting Colorectal Cancer Cell Metabolism with Metformin: An Integrated Transcriptomics Study

**DOI:** 10.3390/cancers16112055

**Published:** 2024-05-29

**Authors:** Ayla Orang, Shashikanth Marri, Ross A. McKinnon, Janni Petersen, Michael Z. Michael

**Affiliations:** 1 Flinders Health and Medical Research Institute, Flinders University, Bedford Park, SA 5042, Australia; ayla.orang@flinders.edu.au (A.O.); shashikanth.marri@flinders.edu.au (S.M.); ross.mckinnon@flinders.edu.au (R.A.M.); janni.petersen@flinders.edu.au (J.P.); 2 Nutrition and Metabolism, South Australia Health and Medical Research Institute, Adelaide, SA 5000, Australia; 3 Department of Gastroenterology and Hepatology, Flinders Medical Centre, Bedford Park, SA 5042, Australia; 4 Flinders Centre for Innovation in Cancer, Flinders Medical Centre, Bedford Park, SA 5042, Australia

**Keywords:** metformin, colorectal cancer, microRNAs, PI3K/Akt pathway, MAPK/ERK pathway

## Abstract

**Simple Summary:**

Metformin, a primary treatment for type 2 diabetes, is known to reduce colorectal cancer (CRC) risk. This study explores the molecular mechanisms behind metformin’s anti-tumour effects in CRC cells. The research identifies specific microRNAs (miRNAs) influenced by metformin, revealing their role in regulating genes associated with cell proliferation and key signalling pathways. The findings provide valuable insights into how metformin disrupts CRC cell growth through post-transcriptional control, impacting both metabolism and cell proliferation.

**Abstract:**

Background: Metformin is a first-line therapy for type 2 diabetes as it disrupts cellular metabolism. Despite the association between metformin and lower cancer incidence, the anti-tumour activity of the drug in colorectal cancer (CRC) is incompletely understood. This study identifies underlying molecular mechanisms by which metformin slows colorectal cancer cell proliferation by investigating metformin-associated microRNA (miRNA) and target gene pairs implicated in signalling pathways. Methods: The present study analysed changes in miRNAs and the coding transcriptome in CRC cells treated with a sublethal dose of metformin, followed by the contextual validation of potential miRNA–target gene pairs. Results: Analyses of small RNA and transcriptome sequencing data revealed 104 miRNAs and 1221 mRNAs to be differentially expressed in CRC cells treated with metformin for 72 h. Interaction networks between differentially expressed miRNAs and putative target mRNAs were identified. Differentially expressed genes were mainly implicated in metabolism and signalling processes, such as the PI3K-Akt and MAPK/ERK pathways. Further validation of potential miRNA–target mRNA pairs revealed that metformin induced miR-2110 and miR-132-3p to target *PIK3R3* and, consequently, regulate CRC cell proliferation, cell cycle progression and the PI3K-Akt signalling pathway. Metformin also induced miR-222-3p and miR-589-3p, which directly target *STMN1* to inhibit CRC cell proliferation and cell cycle progression. Conclusions: This study identified novel changes in the coding transcriptome and small non-coding RNAs associated with metformin treatment of CRC cells. Integration of these datasets highlighted underlying mechanisms by which metformin impedes cell proliferation in CRC. Importantly, it identified the post-transcriptional regulation of specific genes that impact both metabolism and cell proliferation.

## 1. Introduction

Metformin has proven anti-cancer properties in cell lines as it reprogrammes the metabolism of cancer cells. The potential mechanisms of action are mainly associated with activation of AMPK, inhibition of the mTOR pathway, regulating inflammatory responses and promoting cell death in cancer stem cells [1,2,3,4,5]. The inhibitory effects of metformin on colorectal cancer (CRC) have previously been reported at the pre-clinical level, as reduced spontaneous intestinal polyp growth in ApcMin/+mice [6] or sensitisation of CRC cells to other chemotherapeutic agents, which has led to clinical trials evaluating the combinatorial effect of metformin with standard chemotherapeutics [7].

Combination of chemotherapeutics with metformin shows additive responses, which highlights the pleiotropic nature of this drug through simultaneously targeting numerous pathways affecting both tumourigenesis and tumour progression [8,9,10]. Therefore, the molecular mechanisms and crosstalk between signalling pathways that contribute to the anti-cancer effects of metformin remain elusive. Phosphatidylinositol-3-kinase (PI3K)/AKT signalling is an important intracellular pathway and considered a master regulator for cancer [11]. The MAPK/ERK pathway also plays an important role in complex cellular programmes including proliferation, differentiation, development, transformation and apoptosis and has been shown to play a key role in the transduction of extracellular signals to elicit cellular responses [12].

Growing evidence confirms that several miRNA changes are associated with the anti-proliferative activity of metformin. Accordingly, metformin treatment altered miRNA expression in hepatocellular carcinoma, pancreatic cancer, oesophageal squamous cancer, gastric cancer, lung cancer and prostate cancer [13,14,15,16,17].

In general, specific alterations in miRNA expression are likely to result in functional changes in the expression of other genes. Hence, the implications of the interplay between metformin-regulated miRNAs and their target genes, within relevant metabolic and signalling pathways, require further investigation. Given this rationale, an integrated-systems biology approach is necessary to understand the transcriptional and post-transcriptional responses to metabolic disruption in colorectal cancer.

This study provides a comprehensive snapshot of the changes associated with metformin treatment of HCT116 cells, using small RNA sequencing and transcriptome analyses, and characterises metformin response pathways in a systematic manner across different CRC cell lines. It identifies specific miRNA–target transcript interactions that contribute to the metformin response in colorectal cancer cells.

## 2. Materials and Methods

### 2.1. Cell Lines and Reagents

Colorectal cancer cell lines (HCT116, DLD1, RKO, HT29, Caco2 and SW480) were obtained from the American Type Culture Collection and cultivated in Dulbecco Modified Eagle Medium (DMEM) supplemented with 10% Fetal Bovine Serum (FBS). STMN1 siRNA and miRNA mimics (as well as matched negative control siRNA and mimics) were synthesised by Shanghai GenePharma (Shanghai, China), while the PIK3R3 siRNA was purchased from Qiagen (PIK3R3_8 FlexiTube siRNA and #SI03650325 negative control; Valencia, CA, USA). The sequences for these reagents are presented in Supplementary Table S7. Metformin was purchased from Sigma-Aldrich (St. Louis, MO, USA).

### 2.2. Transcriptome and Small RNA Sequencing

To perform Next-Generation Sequencing, the quality of RNA samples was confirmed using an Agilent 2100 Bioanalyzer system (Agilent Technologies, Santa Clara, CA, USA). Following RNA preparation, the generation of libraries, including adapter ligation and PCR amplification, then sequencing were performed at the Flinders Genomics Facility (South Australian Genomics Centre, Adelaide, Australia) using TruSeq Stranded Total RNA and small RNA Sample Preparation Kits (Illumina Inc., San Diego, CA, USA) for RNA and small RNA sequencing, respectively. Ribosomal RNA (rRNA) depletion was performed for RNA sequencing prior to library preparation. The paired-end 100 bp sequencing was performed using the Illumina NextSeq sequencing platform (Illumina Inc., San Diego, CA, USA), and approximately 30–40 and 10–20 million reads were generated per sample for total RNA and small RNA sequencing, respectively.

The QIAseq Targeted RNA Panel (human apoptosis and cell death pathway finder RHS-002Z) from Qiagen (Hilden, Germany) served as a supplementary approach for assessing gene expression. RNA was extracted from HCT116 cells (*n* = 2) subjected to either no treatment (0 mM) or metformin treatment (2.5 mM) over 72 h, as described earlier. Following the manufacturer’s protocol (Qiagen), samples were processed and sequenced using the Illumina MiSeq platform, resulting in approximately 19.5 million reads.

Following quality control, RNA-seq data were trimmed for adaptor sequences using the FASTX-toolkit followed by quality analysis of the reads using FASTQ and assembly, mapping and alignment of reads to the Ensembl human genome (Grch38.p5_v24) or miRbase 20.1 using STAR [18] and SAMtool [19]. Aligned reads were then converted to raw counts using HTseq [20], and differential expression analysis was performed using DESeq2 [21]. The differentially expressed miRNAs and mRNAs were refined using the following criteria: raw counts ≥ 50, adjusted *p* value ≤ 0.05 and −1 ≥ log2 fold change ≥ 1.

### 2.3. microRNA Target Analysis

To predict miRNA targets and collate previously validated targets, seven online resources were used. miRTarBase (release 7.0) [22] and miRecord (release 2013) [23] contain validated miRNA targets, and those targets that were validated with strong evidence (reporter assays) were selected. To collect the predicted targets, TargetScan (release 7.1) [24], miRmap (release 1.1) [25], miRDB (release 6.0) [26], DIANA-microT-CDS (release 5.0) [27] and miRanda (release 3.3a) [28] were used. In TargetScan, targets with a context score smaller or equal to −0.15 were retained. In DIANA-microT-CDS, targets with miTG scores greater or equal to 0.85 were selected. Predicted targets from miRDB with a score greater or equal to 80 were also kept. For miRanda and miRmap, cut-off thresholds of miRSV score ≤ −1.2 and miRmap score ≥ 90 were applied, respectively. Finally, differentially expressed miRNA–target mRNA pairs were extracted only if there was an anti-correlation between metformin-induced miRNA and mRNA levels (if miRNA was downregulated, the mRNA should be upregulated and vice versa) and the pairs were common to at least two databases.

### 2.4. Network Construction and Analysis

Protein–protein interaction (PPI) networks were constructed using the NetworkAnalyst online tool (Release 3.0) and extracted as a zero-ordered network from IMEx Interaction using curated comprehensive datasets through InnateDB [29]. The PPI network was visualised, and the expression values were incorporated into the network using Cytoscape software (version 3.4.0) [30]. The PPI network was then analysed by NetworkAnalyzer, a plug-in of Cytoscape, and the topological properties of the network were assessed [31].

### 2.5. Pathway and Gene Ontology Analysis and Subnetwork Construction

ClueGo (version 2.3.3), a Cytoscape plug-in, was used to extract the non-redundant KEGG (version 305) pathways and gene ontology (GO) terms (Biological Processes, Molecular Functions and Cellular Compartments) in functionally organised networks representing the connection between the pathways or GO terms based on the similarity of their linked genes [32]. At least 3 genes per term and 4 percent attribution in the term were considered. The terms were grouped based on the kappa scores, which is a robust measurement of the correlation occurring by chance. For network connectivity, the kappa score was set at 0.04. GO term fusion was applied, and the cut-off for the term *p* value was 0.05. For GO analysis, “experimental evidence” was selected.

### 2.6. Reverse Transcription Polymerase Chain Reaction

Total RNA was extracted with TRIzol RNA Isolation Reagent (Thermo Fisher Scientific, Waltham, MA, USA) using the standard method. To remove any genomic DNA contamination, the RQ1 RNase free DNase kit (Promega, Madison, WI, USA) was used, and the DNase I enzyme was then deactivated by adding 2.5 µL DNase deactivation slurry from the Ambion DNA-free™ Kit (Invitrogen, Waltham, MA, USA). cDNA was made from 1 µg total DNase-treated RNA with M-MLV Reverse Transcription kit (Promega) and Random Hexamer primers (Invitrogen) according to the manufacturer’s recommended conditions. Real-time RT-PCR reactions were subsequently performed in triplicate using Power SYBR green quantitative real-time RT-PCR master mix (Invitrogen, Life Technologies, Waltham, MA, USA) in a Qiagen Rotorgene Q real-time PCR cycler (Qiagen). Relative expression levels were calculated from Ct values using Qgene [24]. Results were normalised relative to the β-actin (*ACTNB*) expression levels. The PCR primer sequences are listed in Supplementary Table S7.

For miRNA expression analysis, TaqMan miRNA assays (Applied Biosystems, Life Technologies, Waltham, MA, USA) were used (Supplementary Table S7). cDNA was synthesised from 20 ng total RNA using miRNA-specific primers according to the TaqMan miRNA Assay protocol. Real-time PCR was then carried out according to the TaqMan protocol using 1 µL of reverse transcription product, 1× TaqMan Universal PCR Master Mix No AmpErase UNG (Applied Biosystems), miRNA-specific primer and probe assay mix. Results were normalised relative to endogenous miR-16 expression.

### 2.7. Western Blot Analyses

Total extracts were prepared in radioimmunoprecipitation assay buffer (RIPA buffer) containing protease and phosphatase inhibitors (Merck, Darmstadt, Germany). Protein concentration was determined using EZQ^®^ Protein Quantitation Kit (BioRad, Hercules, CA, USA). Samples were then boiled for 3 min at 98 °C, separated in SDS-PAGE gel electrophoresis and transferred to a LF-PVDF membrane (BioRad) using Trans-blot Turbo System (BioRad). The membrane was blocked in TBS with 5% skim milk for an hour and incubated overnight at 4 °C with primary antibodies including α-Actinin (Cell Signaling Technology, Danvers, MA, USA, 1:1000), PIK3R3 (Cell Signaling Technology, 1:1000), STMN1 (Abcam, Cambridge, UK, 1:10,000), p70 S6K (Cell Signaling Technology, 1:1000) and Phospho-p70 S6K (Thr389-Cell Signaling Technology, 1:500) followed by incubation for 1 h with anti-rabbit HRP-conjugated secondary antibody at a dilution of 1:20,000. ECL assay was used to visualise bands using ChemiDoc™ Imaging Systems (BioRad) and analysed using AlphaEaseFC™ software (Alpha Innotech, San Leandro, CA, USA, http://genetictechnologiesinc.com/alpha/alpha_ease_fc.htm (accessed on 17 May 2024)).

### 2.8. Cell Viability Assays

Crystal violet solution was made by dissolving 100 mg crystal violet (Sigma-Aldrich) in 500 mL 10% buffered formalin (Orion Labs, San Francisco, CA, USA). To perform the assay for cells grown in 96-well plates, the plate was washed with 1× PBS. A 50 µL amount of 1× crystal violet solution was added to each well and incubated at room temperature for 10 min. The plate was then washed, the crystal-violet-stained DNA was solubilised in 100 µL 1% SDS solution and the absorbance at 570 nm was measured using an EnSight Multimode Plate Reader (Perkin Elmer, Shelton, CT, USA).

### 2.9. Flow Cytometry Analyses

To define the cell cycle distribution, flow cytometric analyses were performed. In brief, cells grown in 6-well plates were harvested by trypsinisation and fixed with 80% ethanol. Cells were stained for total DNA content with a solution containing 50 µg/mL propidium iodide and 200 µg/mL RNase I and 0.1% Triton X-100 in PBS for 30 min. Cell cycle distribution was then assayed with a CytoFLEX flow cytometer (Beckman Coulter Life Sciences, Brea, CA, USA) and the data analysed using FCS Express (De Novo Software, Pasadena, CA, USA, https://denovosoftware.com/ (accessed on 17 May 2024)).

### 2.10. miRNA Target Validation

miScript Target Protectors (Qiagen) were used to validate predicted miRNA::mRNA target pairs and binding regions (Supplementary Table S7). Target protectors are nuclease-resistant, single-stranded oligonucleotides that competitively hybridise with miRNA binding sites on the 3′UTR of the predicted targets while having no effect on other targets of the same miRNA [33]. The Qiagen online algorithm was used to design target protectors. In 24-well plates, HCT116 cells were co-transfected with miRNA mimics (20 nM) and also with the corresponding target protector/s (500 nM) designed for the specific transcript binding sites, or with a negative control target protector (Qiagen: MTP0079571, MTP0079585, MTP0079592, MTP0079578, MTP0079599 and MTP0000002), for 72 h followed by RT-PCR or crystal violet assays.

## 3. Results

### 3.1. Metformin Treatment of Colorectal Cancer Cells Induces Integrated miRNA::mRNA Responses

**Figure 1:**
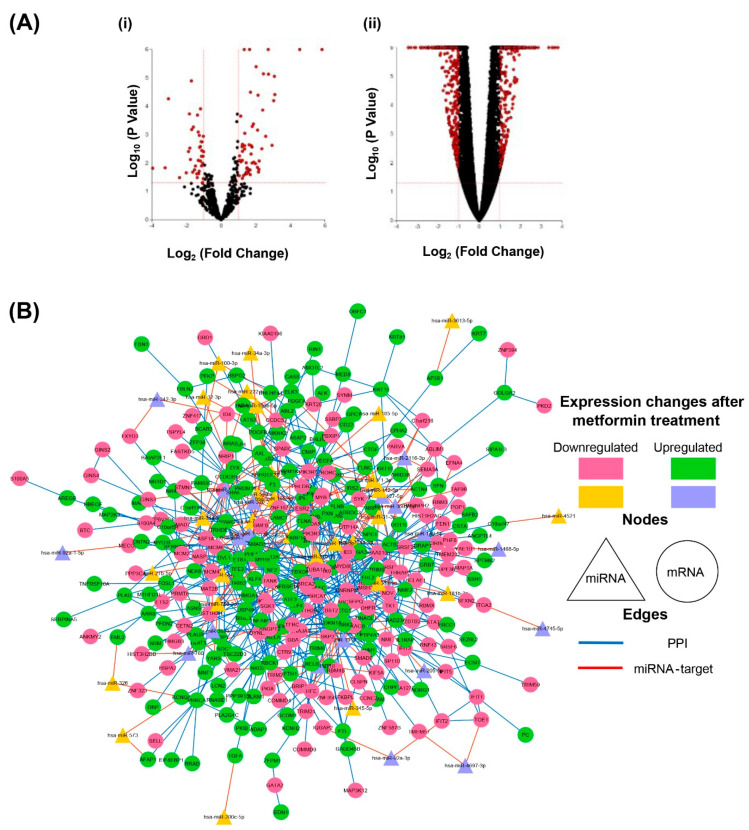
Metformin induces gene and miRNA expression changes affecting miRNA-based networks in colorectal cancer cells. (**A**) Volcano plots for transcriptome and small RNA profiling of HCT116 cells treated with 2.5 mM metformin are shown. (i) Protein-coding genes and (ii) miRNAs that were identified by sequencing are shown. Red dots represent differentially expressed genes/miRNAs, while those in black represent no significant changes. Advaita iPathway Guide tool was used to generate the plot. Y axis represents log10 (*p* value), and x axis represents log2 fold change. Differentially expressed mRNAs and miRNAs were identified with thresholds set at *p* ≤ 0.05 and 1 ≤ log2 fold change ≤ −1. (**B**) miRNA-based network affected by metformin treatment of colorectal cancer cells is shown. This is an organic layout of the integrated network associated with 2.5 mM metformin treatment of HCT116 cells. Protein–protein interactions are from the InnateDB signalling network, and miRNA–target pairs are collated from validated and predicted databases. miRNAs are shown as triangles, and genes are the circular nodes. The miRNA–gene interactions are shown as red lines, while gene–gene interactions are blue. The pink and orange nodes represent downregulated genes and miRNAs, respectively, while green and purple nodes are upregulated genes and miRNAs, respectively.

To investigate the altered miRNA and transcriptome profiles resulting from metformin treatment at a sublethal dose (2.5 mM) in HCT116 colorectal cancer cells, small RNA-seq and total RNA-seq were performed. The 2.5 mM metformin concentration was chosen as it reproducibly slows HCT116 proliferation by ~20% over 72 h, without inducing apoptosis [34]. This concentration is also physiologically relevant in the gut epithelium [35]. Differential expression analysis identified 104 miRNAs, with 63 and 41 miRNAs being upregulated or downregulated, respectively (Figure 1 and Supplementary Table S1). Also, 1221 differentially expressed (DE) protein-coding genes were identified, with a total of 554 upregulated and 667 downregulated genes (Figure 1(Aii) and Supplementary Table S2). The data reliability was confirmed by comparing the common differentially expressed genes between the total RNA-seq (TruSeq Stranded Total RNA) and QIAseq Targeted RNA Panel, and the strong positive correlation (r = 0.86) showed the reproducibility and reliability of the total RNA-seq results (Figure S1, Supplementary Table S3).

To construct an integrative network including DE miRNAs and genes, miRNA target gene prediction was performed as detailed in Section 2. The validated and predicted protein-coding gene targets of 104 DE miRNAs were then collected. Relatively strict thresholds were selected in an attempt to reduce the number of false-positive targets. The potential DE miRNA–gene pairs identified showed anti-correlations in their expression changes and were common to two or more prediction databases. To ensure the robustness of protein-coding gene identification, the genes with fewer than six reads per million in either metformin-treated or untreated RNA sequencing data were excluded. A total of 1060 DE genes were thus assigned for the following analyses. In total, 167 miRNA::mRNA pairs were identified, with only 10% of predictions having been experimentally validated (Table S4). Using protein–protein interactions together with miRNA–gene interactions (predicted or validated), a miRNA-based network was then assembled (Figure 1B). The integrated miRNA-based network compromised 365 nodes (47 miRNAs, 317 mRNAs) and 728 edges (169 miRNA–gene interactions and 559 protein–protein interactions). Overall, this network demonstrates the complex changes in miRNA–gene and gene–gene interactions. Since DE miRNAs potentially target multiple and highly connected genes within the integrated network, these miRNAs together with putative/validated target genes represent complex metformin-mediated biochemical pathway changes in HCT116 cells.

### 3.2. Metformin-Associated Differentially Expressed miRNAs and Genes Are Enriched in PI3K-Akt and MAPK/ERK Signalling

**Figure 2:**
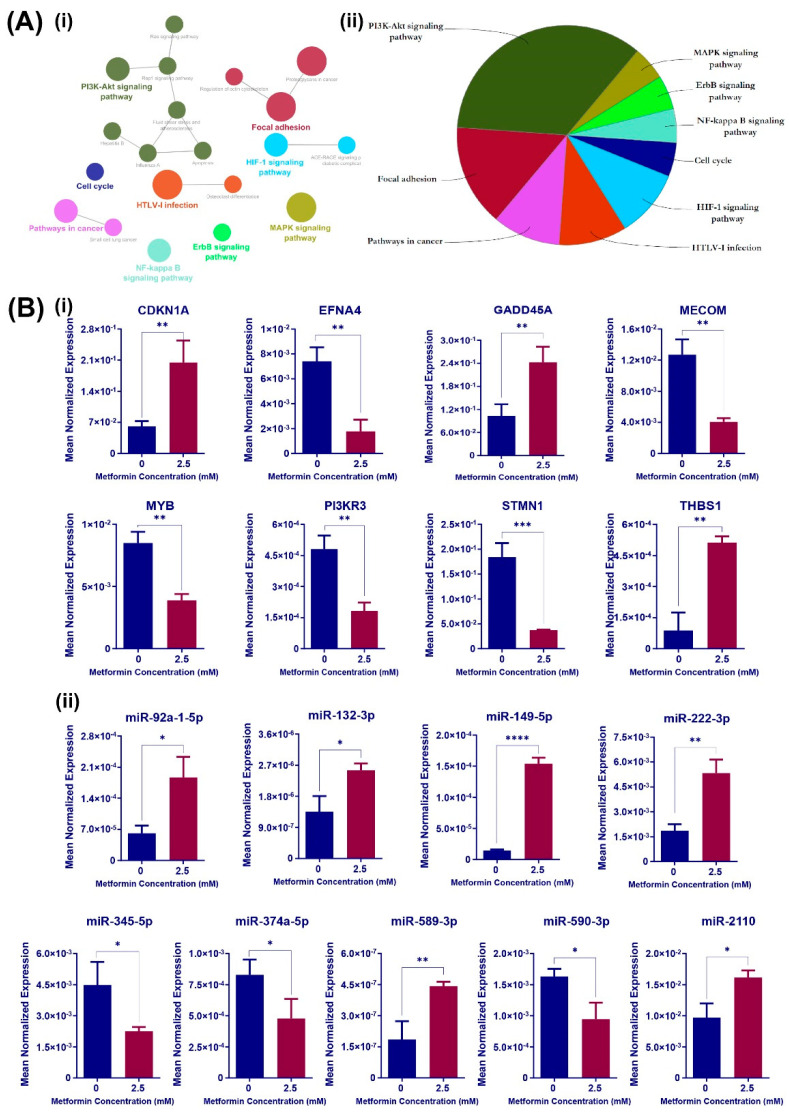
Differentially expressed genes and miRNAs associated with metformin treatment of HCT116 cells are overrepresented in PIK3-AKT and MAPK signalling pathways. (**A**) Functionally grouped network of enriched categories was generated for all the differentially expressed genes with degree ≥ 1 by querying the KEGG database using ClueGO plug-in. Pathways are represented as nodes, and the node size represents the term’s enrichment significance (i). The node pie chart represents the KEGG pathway analysis of selected genes. Only the most significant term in the group is labelled, and the size of each category within the pie chart represents the number of included terms (ii). (Term *p* value corrected with Bonferroni step down ≤ 0.05) (**B**) Real-time PCR analyses of relative mRNA (i) and miRNA (ii) levels in HCT116 cells for genes identified in PI3K-Akt and MAPK signalling pathways as being influenced by 2.5 mM metformin treatment are shown. Cells were treated with 2.5 mM metformin for 72 h and compared with cells in control medium. Results are expressed as mean ± SD of at least 3 replicates, and the expression levels of selected mRNAs and miRNAs were normalised to B2M and miR-16 expression levels, respectively. Statistical significance is indicated with asterisks (* *p* ≤ 0.05, ** *p* ≤ 0.01, *** *p* ≤ 0.001, **** *p* ≤ 0.0001).

To examine which biological pathways were altered in HCT116 cells treated with 2.5 mM metformin, the biochemical pathways were extracted from the Kyoto Encyclopedia of Genes and Genomes (KEGG) collection, the DE genes with at least one interaction were extracted from the integrative network (Figure 1B) and the ClueGO Cytoscape plug-in was used for further analyses. KEGG pathway analysis revealed 20 statistically enriched pathways organised into eight groups based on kappa score (Figure 2A). “PI3K-Akt signalling pathway” had the lowest *p* value of all GO groups and was the parent term within this group (*p* = 1.0 × 10^−8^). In addition, “MAPK/ERK signalling pathway” had the lowest term *p* value (*p* = 7.4 × 10^−7^), with 25 DE genes involved in this pathway (Table S4).

The involvement of miRNAs in the PI3K-Akt and MAPK/ERK signalling pathways was investigated by identifying potential miRNA::target gene pairs within the two cascades (Table S6). After applying a further stringent selection criterion by filtering out miRNAs with fewer than 12 reads per million and retaining those miRNA::gene pairs if downregulated, miRNAs were described as oncogenic miRNAs, and their putative upregulated target genes had a tumour suppressor role in cancer; vice versa, six (four predicted and two validated) and four (three predicted and one validated) miRNA::mRNA pairs remained in the PI3K-Akt or MAPK/ERK signalling pathways, respectively, as shown in Table S6. RT-PCR validation of the expression changes in miRNAs and genes associated with 2.5 mM metformin treatment was then performed in an independent experiment (Figure 2B). These results collectively represent the multi-functional responses to metformin in altering miRNA profiles of CRC cells to fine-tune associated gene interactions and consequent signalling pathways.

### 3.3. Metformin-Induced miRNAs Directly Target Key PI3K-Akt and MAPK/ERK Signalling Components

**Figure 3:**
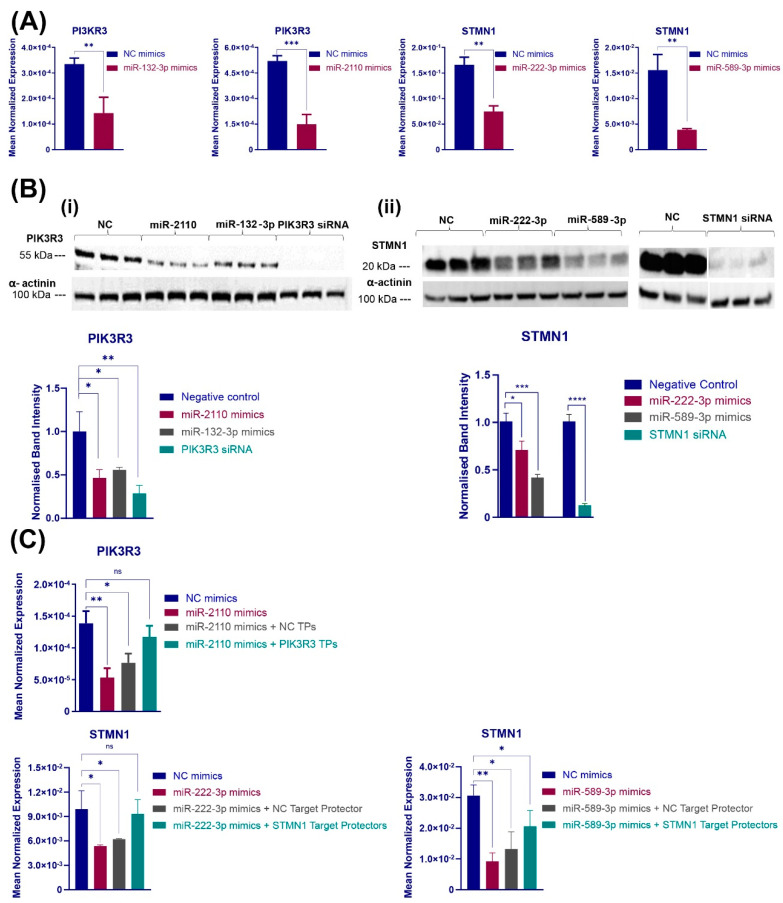
Metformin-associated differentially expressed miRNAs directly target molecular components of PI3K-AKT and MAPK signalling pathways. (**A**) Gene expression changes associated with exogenous overexpression of selected miRNA were assessed by real-time RT-PCR and compared with NC mimic. Results are expressed as mean ± SD of at least 3 replicates, and expression levels were normalised to *ACTB* levels. Statistical significance is indicated with asterisk (** *p* ≤ 0.01, *** *p* ≤ 0.001). (**B**) PIK3R3 (i) and STMN1 (ii) protein levels and densitometry analysis in cells transfected with miR-132-3p/miR-2110/NC mimic and miR-222-3p/miR-589-3p/NC mimic are shown, respectively, as measured by Western blot analysis 76 h post-transfection. Results are expressed as mean ± SD of 3 replicates, and statistical significance is indicated with asterisks (* *p* ≤ 0.05, ** *p* ≤ 0.01, *** *p* ≤ 0.001 and **** *p* ≤ 0.0001). (**C**) Validation of direct targeting of PIK3R3 and STMN1 by corresponding miRNAs is shown. mRNA levels of miR-2110 target gene (PIK3R3) and miR-222-3p and miR-589-3p target gene (*STMN1*) in HCT116 cells co-transfected with miRNA mimics or NC mimics and with NC target protectors or PIK3R3/STMN1 target protectors for 72 h and normalised to *ACTNB* expression levels are shown. Results are expressed as mean ± SD of 3 replicates, and statistical significance is indicated with asterisks (ns *p* > 0.05, * *p* ≤ 0.05, ** *p* ≤ 0.01).

To validate direct or indirect targeting of genes by selected miRNAs within PI3K-Akt and MAPK/ERK signalling pathways, HCT116 cells were transfected with corresponding synthetic miRNA mimics, and the putative target transcript levels were assessed by RT-PCR. Among the selected miRNA::mRNA pairs in the PI3K-Akt pathway, exogenous introduction of miR-132-3p or miR-2110 activity resulted in a significant reduction in *PIK3R3* gene expression of more than 60% (*p* = 0.0073 and 0.0006, respectively), as shown in Figure 3A. Also, *THBS1* gene expression was downregulated as a result of miR-590-3p mimic transfection (*p* = 0.0029, Figure S2A), while there was no change in expression levels for the predicted targets *MYB*, *EFNA4* and *CDKN1A* associated with the transfection of miR-132-3p, miR-149-3p and miR-345-5p mimics, respectively (Figure S3). In addition, among four putative/validated miRNA::mRNA pairs within the MAPK/ERK signalling pathway, miR-222-3p and miR-589-3p transfection both resulted in *STMN1* gene expression downregulated by 56% and 73%, respectively (Figure 3A), while there was no significant change in expression of *MECOM* and *GADD45A* in cells transfected with miR-92a-1-5p and miR-374a-5p, respectively (Figure S3). The miR-590-3p::*THBS1A* pair was excluded from following investigations as increasing miR-590-3p activity in HCT116 cells did not significantly affect cell proliferation (Figure S2).

To further explore miR-2110 and miR-132-3p targeting *PIK3R3* as well as miR-222-3p and miR-589-3p targeting *STMN1*, the protein levels of the corresponding targets were assessed in cells transfected with miRNA mimics. PIK3R3 and STMN1 immunoblots showed a prominent decrease in the normalised band intensity of the putative/validated target genes in cells transfected with the corresponding miRNAs compared to the cells transfected with scrambled negative control (NC) mimics and normalised to α-actinin band intensity. Accordingly, cells transfected with miR-2110 or miR-132-3p mimics, or a specific PIK3R3 siRNA, showed an at least 50% reduction in PIK3R3 protein levels (Figure 3(Bi)). Additionally, as shown in Figure 3(Bii), transfection of HCT116 cells with miR-222-3p and miR-589-3p precursors led to a 30% and 60% reduction in normalised STMN1 protein band intensity, respectively, and diminished expression of STMN1 at protein levels observed with transfection of STMN1 siRNA. These results therefore confirm the direct targeting of *PIK3R3* by miR-132-3p, as described in [36]. They also suggest a role for miR-2110 in the regulation of *PIK3R3* as well as both miR-222-3p and miR-589-3p in the regulation of *STMN1* in HCT116 cells.

*PIK3R3* is a validated target of miR-132-3p [28], and, as listed in Table S6, it is identified as a novel target of miR-2110 by TargetScan and miRmap prediction algorithms. Based on the target predictions, the 3′-UTR of *PIK3R3* mRNA contains three potential binding sites for miR-2110 (Figure 3C). Similarly, miR-222-3p and miR-589-3p were predicted to have binding sites on the 3′UTR of *STMN1* mRNA by microT-CDS, miRanda and TargetScan algorithms (Table S6). To confirm the direct binding of miR-2110 to the *PIK3R3* 3′UTR as well as miR-222-3p and miR-589-3p to predicted sites in the *STMN1* 3′UTR, miScript Target Protectors were designed to obstruct miRNA interaction with the specific transcript binding sites [37]. HCT116 cells were co-transfected with miR-2110 mimics and a combination of three target protectors, each designed to individual binding sites in the *PIK3R3* 3′UTR, as well as control transfections with negative control (NC) mimics and/or a negative control (NC) target protector. miR-2110 mimics alone and miR-2110 mimics with NC target protectors resulted in a 62% and 45% reduction in *PIK3R3* transcript levels, respectively (*p* = 0.0035 and 0.0111, respectively), as shown in Figure 3C. Introduction of the NC target protector did not protect PIK3R3 from miR-2110 binding and regulation (*p* = 0.4423), as shown in Figure 3C. *PIK3R3* RT-PCR showed an increase in cells co-transfected with miR-2110 mimics and *PIK3R3* 3′UTR target protectors compared with the cells co-transfected with miR-2110 mimics and NC target protectors, as well as with the cells transfected with miR-2110 mimics alone (0.5- and 1.2-fold increase, respectively), as shown in Figure 3C. In addition, HCT116 cells were co-transfected with miR-589 or miR-222-3p mimics and the corresponding *STMN1* 3′UTR target protectors as well as negative control treatments with NC mimics and/or NC target protectors. miR-222-3p and miR-589-3p mimics alone resulted in a 46% and 70% reduction in *STMN1* gene expression, respectively (*p* = 0.025 and 0.0012, Figure 3C), while NC target protectors had little to no effect on the miRNA-associated STMN1 downregulation (*p* = 0.102). Co-transfection of miR-222-3p with the corresponding *STMN1* site target protector almost completely negated the silencing effect of the miRNA mimic, and miR-589-3p co-transfection with its corresponding STMN1 target protector restored *STMN1* expression by 58% compared with mimics combined with the NC target protector and a 124% increase compared with the mimics alone (Figure 3C). Together, these results confirm the prevention of miRNA binding to the corresponding specific binding sites in the 3′UTRs of *PIK3R3* or *STMN1* mRNAs by specific target protectors. They also support the findings that *PIK3R3* and *STMN1* transcripts are direct targets of miR-2110 or miR-222-3p and miR-589-3p-mediated repression, respectively.

### 3.4. Metformin-Associated miRNAs That Target PI3K-Akt and MAPK/ERK Signalling Pathways Also Suppress Colorectal Cancer Cell Proliferation and Induce Cell Cycle Arrest

**Figure 4:**
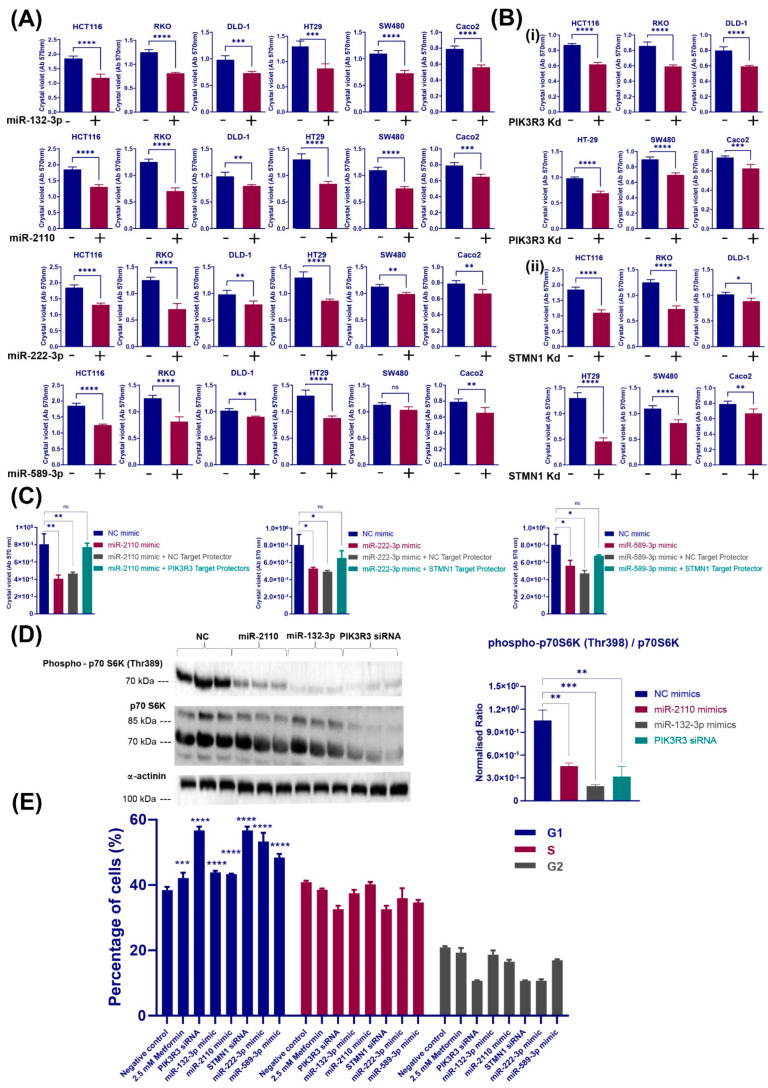
Metformin-associated differentially expressed miRNAs regulate PI3K-AKT and MAPK signalling pathways to suppress cell proliferation and induce cell cycle arrest. (**A**) Proliferation of CRC cells 96 h post-transfection with miR-132-3p, miR-2110, miR-222-3p and miR-589-3p mimics is shown. Quantitative results of crystal violet staining of transfected HCT116, RKO, SW480, HT29, DLD1 and Caco2 cells confirmed the reduction in cell proliferation with miR-132-3p, miR-2110 and miR-589-3p mimic transfection. “−” denotes a NC mimic, while “+” denotes a specific miRNA mimic. (**B**) Proliferation of different colorectal cancer cell lines 96 h after transfection with PIK3R3 (i) or STMN1 (ii) siRNA is shown. Quantitative results of crystal violet staining of transfected HCT116, RKO, SW480, HT29, DLD1 and Caco2 cells validated the suppression of cell proliferation following *PIK3R3* or *STMN1* gene knockdown. “−” denotes NC siRNA, while “+” denotes gene-specific siRNA. Statistical significance is indicated with asterisks (ns *p* > 0.05, * *p* ≤ 0.05, ** *p* ≤ 0.01, *** *p* ≤ 0.001, **** *p* ≤ 0.0001). (**C**) Cell viability measurements using crystal violet assays in HCT116 cells co-transfected with STMN1 or PIK3R3 target protectors with corresponding miRNA mimics or NC mimics and with negative control target protector for 72 h. Results are expressed as mean ± SD of at least 5 replicates, and statistical significance is indicated with asterisks (ns *p* > 0.05, * *p* ≤ 0.05, ** *p* ≤ 0.01). (**D**) Alteration in p70 S6 kinase phosphorylation levels of HCT116 cells after transfection with miR-132-3p, miR-2110 mimics or PIK3R3 siRNA compared to NC is shown. Densitometry results were normalised against corresponding α-actinin protein levels. Results are expressed as mean ± SD of 3 replicates, and statistical significance is indicated with asterisk (** *p* ≤ 0.01, *** *p* ≤ 0.001). (**E**) Cell cycle distribution of HCT116 cells after transfection with miRNA mimics, PIK3R3 and STMN1 siRNA or metformin treatment for 96 h are shown. Histograms show the percentage of HCT116 cells in different cell cycle phases following transfection with miR-132-3p, miR-2110, miR-222-3p, miR-589-3p miRNA mimics, PIK3R3 and STMN1 siRNAs or treatment with 2.5 mM metformin. Results are expressed as mean ± SD of 3 replicates compared to the negative control, and statistical significance is indicated with asterisks (*** *p* ≤ 0.001, **** *p* ≤ 0.0001).

To investigate the roles of validated miRNA–gene pairs (miR-132-3p and miR-2110 targeting *PIK3R3*, miR-222-3p and miR-589-3p targeting *STMN1*) in cellular processes, such as cell proliferation and cell cycle progression, in different CRC cell models, miRNA mimics were transfected into a panel of CRC cell lines, then viability assayed using crystal violet assay. In addition, the percentage of HCT116 cells at each cell cycle stage was assessed by flow cytometry. HCT116, RKO, DLD1, HT29 and Caco2 cell transfections with miR-132-3p, miR-2210, miR-222-3p or miR-589-3p mimics resulted in decreased cell proliferation. Among these lines, HCT116, RKO and HT29 showed the lowest cell viability, an up to 45% decrease, with each of the overexpressed miRNAs compared with DLD1, Caco2 and SW480 cells (Figure 4A). To confirm that target genes *PIK3R3* and *STMN1* are important in CRC, the role of *PIK3R3* and *STMN1* genes in regulating cell proliferation of CRC cell lines was examined. Crystal violet assays showed that RNA interference with PIK3R3 and SMTN1 siRNAs significantly decreased growth, up to a 35% and 65% reduction, respectively, compared with a negative control siRNA transfection at 72 h post-transfection in all six different colorectal cell lines (Figure 4B). The consistent decrease in CRC cell proliferation following *STMN1* and *PIK3R3* knockdown confirms their pro-proliferative roles across CRC cell lines.

To investigate the biological relevance of the specific interactions between *PIK3R3* or *STMN1* and their corresponding miRNAs, cell proliferation was examined following the co-transfection of HCT116 cells with PIK3R3 or STMN1 target protectors and miRNA mimics. As shown in Figure 4C, cell viability measurements using crystal violet assays demonstrated that the restoration of *PIK3R3* or *STMN1* mRNA levels by target protectors, where specific miRNA binding is prevented, increases the viability of HCT116 cells compared with the cells transfected with miRNA mimics alone. The viability increases detected were 40% with miR-2110 target protectors, 15% with miR-222-3p target protectors and 30% with miR-589-3p target protectors. Collectively, these results indicate that *PIK3R3* or *STMN1* silencing by the validated miRNAs contributes to the anti-proliferative effect of these miRNAs in CRC cell lines. They confirm the tumour suppressor roles of the selected miRNAs as inhibitors of cell proliferation.

To further confirm the cellular changes associated with *PIK3R3* targeting by miR-2110 and miR-132-3p within the PI3K/Akt pathway, and since the suppression of PI3K/Akt/mTOR signalling is considered a common cellular response to metformin treatment [38,39], the phosphorylation of p70 S6 kinase was assessed as a downstream effector of this pathway [40]. As shown in Figure 4D, transfection of HCT116 cells with miR-132-3p or miR-2110 mimics, as well as *PIK3R3* RNA interference, resulted in a prominent reduction in p70 S6 kinase phosphorylation (Thr389) compared to the negative control. While PIK3R3 siRNA transfection resulted in a slight reduction in total p70 S6K protein levels, miR-2110 and miR-132-3p transfection had no significant effect on p70 S6K levels (Figure 4D).

Cell cycle analysis was performed to examine whether changes in cell cycle control contribute to the reduced cell proliferation that is induced by miRNA-suppressed PIK3R3 or STMN1 activity. Metformin treatment resulted in an increased proportion of cells in G1 phase compared to vehicle-treated HCT116 cells, which indicates partial G1 cell cycle arrest (Figure 4E). Likewise, transfection with STMN1 or PIK3R3 siRNAs, miR-222-3p or miR-589-3p mimics enhanced the percentage of cells in G1 phase compared to vehicle-treated cells (Figure 4E). These data suggest that metformin can partially delay cell cycle progression, mainly at the G1 phase, by inducing these miRNAs and thereby inhibiting their target genes, *PIK3R3* and *STMN1*.

## 4. Discussion

Metformin was shown to alter the expression of 1221 mRNAs and 104 miRNAs in HCT116 cells. Aberrant expression of mRNAs and miRNAs contributes to tumour progression in the gastrointestinal tract, while expression profiles also differ along the gut [41,42,43,44]. While many oncogenic miRNA levels decreased in colorectal cell lines in response to metformin, other miRNAs were shown to increase with metformin treatment. Udhane et al. performed a detailed transcriptome analysis of metformin-associated changes and identified 14 DE genes that are involved in intracellular metabolic processes in polycystic ovaries (PCO) [45].

Metformin reaches serum concentrations of approximately 40 µM in diabetic patients [46]. However, it undergoes substantial accumulation within the human intestinal lining, where concentrations are markedly higher. Studies examining metformin levels extracted from cleansed intestinal biopsies of diabetic individuals have revealed concentrations up to 300 times greater than those found in the plasma [35,47]. Consequently, the elevated levels of metformin within the intestine suggest potential differences from its effects elsewhere in the body.

Our study of the genome-wide impact of metformin exposure, with unique KEGG pathway signatures, confirmed that components of PI3K-Akt-related pathways are major molecular targets in HCT116 cells. Further validation of the potential miRNA–target gene pairs affected by metformin treatment and involved in the PI3K-Akt pathway identified *PIK3R3* as a putative target of miR-2110 and miR-132-3p and one of the key regulators of the pathway. miR-2110 and miR-132-3p were significantly upregulated in metformin-treated HCT116 cells. While several studies have highlighted a key onco-suppressive role for miR-132-3p, to date, there is little research on the role of miR-2110 in cancers. Regardless, miR-2110 has been shown to be rectal cancer specific and to be associated with tumour development [48,49]. It is a neurite-inducing miRNA, shown to exert a pro-differentiation and tumour-suppressive role in neuroblastoma [50]. Similarly, Zhao et al. showed the anti-cancer effect of miR-2110: inducing neuroblastoma cell differentiation and reducing cell survival through targeting Tsukushi (TSKU) [51]. Another study on neuroblastoma showed a similar effect of miR-2110 and the inverse expression of miR-2110 with *MYCN* mRNA levels [52]. Ectopic expression of miR-132-3p was shown to significantly inhibit CRC cell proliferation and invasion, and to be correlated with increased sensitivity to preoperative chemoradiotherapy [53,54,55]. Zheng et al. also showed the inhibitory effect of miR-132-3p on CRC cell invasion and metastasis through direct targeting of *ZEB2* [56]. Downregulation of this miRNA by DNA hypermethylation has also been associated with CRC invasion and poor prognosis in colorectal cancer [54,57]. miR-132-3p suppression of CRC cell proliferation, migration and invasion, and induced apoptosis, has also been accredited with targeting transcripts *Derlin-1* and *CREB5* [58,59]. As shown in this study, metabolic disruption of HCT116 cells with 2.5 mM metformin resulted in a significant increase in miR-132-3p and miR-2110 expression levels, and a significant reduction in *PIK3R3* expression.

The PI3K intracellular signalling pathway plays a critical role in cell apoptosis, cell cycle progression, proliferation and protein synthesis. Its role in regulating glucose uptake and metabolism is equally definitive. PI3K dysregulation was reported in several human cancers, and drugs targeting this pathway are currently in clinical trials [60]. An overactivated PI3K-Akt pathway, caused by gene mutations and/or amplifications, is commonly associated with tumourigenesis. Activation of PI3K leads to the induction of downstream effectors such as AKT and mTOR [61].

Liu et al. confirmed direct *PIK3R3* targeting by miR-132-3p in hepatocellular carcinoma. They also showed that the expression levels of *PIK3R3* were significantly downregulated in liver cancer tissues and cell lines compared to normal counterparts and were inversely correlated with tumour differentiation, cancer stage and lymph node metastasis. Investigating the molecular target of miR-132-3p, this study confirmed that suppressing cell proliferation, migration, invasion and inhibition of the Akt/mTOR signalling pathway could be attributed to *PIK3R3* silencing by this miRNA [36].

In our study, we confirmed that enhanced activity of miR-2110 and miR-132-3p resulted in a significant reduction in the proliferation of all six cell lines tested, representing various classifications of CRC. This was accompanied by decreased *PIK3R3* mRNA and protein levels, as well as decreased mTOR signal activation in HCT116 cells, as detected by suppressed p70 S6K phosphorylation. The functional roles of *PIK3R3* in CRC, to sustain cell proliferation (and presumably tumour growth), and the mTOR pathway were both disrupted following *PIK3R3* knockdown, possibly reflecting the consequences of reduced *PIK3R3* levels following metformin treatment. The current study also confirmed *PIK3R3* as a direct target of miR-2110 using target protector assays, verifying direct interaction between miR-2110 and the *PIK3R3* 3′-UTR. Furthermore, the biological function of this targeting, in this case, CRC cell proliferation, was also confirmed.

Through further investigation of likely metformin-associated roles in CRC cells, informed by KEGG pathway analysis, this study revealed that MAPK/ERK signalling had the highest term significance among 20 terms identified to be relevant. Following further validation experiments, miR-222-3p/miR-589-3p::*STMN1* pairs were highlighted as a putative MAPK effector that may contribute to the metformin response.

The MAPK/ERK pathway regulates different cellular characteristics such as gene expression, cell cycle, metabolism, motility, cell survival, apoptosis and differentiation [62]. Both pro- and anti-oncogenic functions have been attributed to miR-222-3p in different cancers, suggesting a context-dependent and bimodal role for this miRNA [63,64,65]. While upregulation of miR-222-3p was reported in some cancers including glioblastoma, non-small-cell lung cancer, lymphoma, Kaposi sarcoma and hepatocellular cancer, in some cases, miR-222-3p was downregulated compared with non-cancer specimens and cells [66,67,68,69]. Functioning as an oncogenic miRNA, miR-222-3p was shown to target *p27Kip1* and *p57* to diminish CDK inhibition. It also induced EMT by noncanonically upregulating *ZEB2* expression [70,71,72]. In contrast, miR-222-3p overexpression in malignant glioblastoma cells increased the cell population in the S phase and induced apoptosis [73]. Fuse et al. reported a reduced level of miR-222-3p in clinical specimens of prostate cancer compared with non-cancer tissues and a tumour-suppressive function for this miRNA in suppressing cell proliferation, migration and invasion [74]. In lung cancer cell lines, growth suppression was also shown through intra-S phase arrest and induced apoptosis [75]. Similarly, phorbol myristate acetate (PMA)-induced overexpression of miR-222-3p in acute myeloid leukemia (AML) resulted in cell cycle arrest and partial differentiation [76].

Whilst some research has been reported for miR-589-5p, there is still little understanding of its function in cancers. Cesarini et al. reported A-to-I editing within the miR-589-3p seed sequence, exclusively in normal brain, but a significant decrease in editing in glioblastoma cell lines and tissue. They also showed suppressed cell proliferation, migration and invasion once miR-589-3p editing was induced in glioblastoma cells [77]. Also, miR-589-3p was shown to promote lumbar disc degeneration (LDD) by playing a pro-apoptotic role in lipopolysaccharide (LPS)-stimulated nucleus pulposus (NP) cells [78].

*STMN1* encodes stathmin1/oncoprotein-18, which is a cytosolic protein that regulates microtubule dynamics during mitotic spindle formation [79,80]. *STMN1* is highly expressed in different types of cancers such as colorectal, gallbladder, breast cancer, hepatocellular carcinoma, sarcoma, lung and prostate adenocarcinomas [81,82,83,84,85,86,87,88]. *STMN1* expression is correlated with the clinical outcome of patients with breast cancer, glioma and hepatocellular carcinoma [89,90,91]. STMN1 is associated with MAPK/ERK signalling and thus can be involved in cell cycle progression [92]. Its involvement in the development of various cancers has been reported [93,94,95]. *STMN1* silencing resulted in inhibited cell growth, induced apoptosis and delayed G2/M phase transition in gallbladder cancer cells by regulating the activity of p38 MAPK kinase and p53/p21 signal pathways [81]. MAPK activity suppresses miR-193b, derepressing STMN1 in pancreatic cancer cells and thereby enabling proliferation [96]. STMN1 was shown to suppress mesenchymal cell motility and correlate with the metastatic phenotype in human sarcomas in vivo [97]. In colorectal cancer, *STMN1* expression is also correlated with elevated cell proliferation and poorer patient prognosis in metastatic disease [94]. As a downstream effector of PI3K/Akt signalling, STMN1 activity contributes to metabolism-related carcinogenesis and is a likely target for therapeutic intervention.

As shown in this study, the expression of both miR-222-3p and miR-589-3p was dramatically increased by metformin treatment in HCT116 cells, and there was a significant reduction in *STMN1* gene expression in treated cells compared with in the control group. Furthermore, the exogenous expression of miR-222-3p and miR-589-3p also reduced CRC cell proliferation and *STMN1* expression at both the mRNA and protein levels. Target protector assays confirmed the direct binding and post-transcriptional suppression of *STMN1* expression by these miRNAs. The biological consequences of *STMN1* targeting by miR-222-3p and miR-589-3p were also confirmed, as direct *STMN1*::miRNA binding was required to significantly suppress CRC cell proliferation.

Cell cycle arrest is often associated with anti-cancer drug activity. Consistent with previous reports, the current study confirmed the role of metformin in suppressing CRC cell proliferation by increasing the proportion of cells in G1 phase. As shown in this study, elevating miR-132-3p and miR-2110 activities, and also miR-589-3p and miR-222-3p activities, resulted in a delayed cell cycle at the G1 phase, which is partly due to direct transcript binding and downregulation of *PIK3R3* or *STMN1* expression, respectively. These miRNAs that are prevalent in various cancers emerge here as central players influenced by metabolic regulation. While our study sheds light on their role in CRC, the broader implications of the metabolic regulation of these miRNAs in other tumour types remain to be fully explored. Further investigation into how metabolic disruption impacts other tumours may unveil novel therapeutic avenues for diverse cancer types.

While our initial transcriptomic analysis was centred on a specific CRC cell line that represents tumours with microsatellite instability, it is noteworthy that the findings were validated across various CRC cell lines with differing genotypes. Such replication across different cellular contexts not only enhances the robustness and reliability of our results but also underscores the generalisability of the identified mechanisms.

A notable limitation of our study is that the experiments were conducted using a high-glucose medium. While this medium is commonly used for cell culture, it does not fully represent the physiological nutrient environment encountered by cells in vivo. Therefore, to better mimic the conditions encountered by tumours in situ, it would be valuable to replicate our experiments using media that more closely resemble physiological nutrient levels.

Altogether, these results combined with previous studies conclude that metformin exerts its anti-cancer properties in part through inducing miRNAs such as miR-2110, miR-132-3p, miR-222-3p and miR-589-3p to directly target genes including *PIK3R3* and *STMN1*, and thereby regulates signalling pathways such as PI3K-Akt and MAPK/ERK.

While the roles of PIK3R3 and STMN1 and their regulation are well described, this study uncovers a novel mode of regulation of these genes at the post-transcriptional level. These findings highlight the potential for developing RNA therapeutics. Moving forward, further investigations are warranted to elucidate the clinical implications and therapeutic opportunities stemming from these discoveries, paving the way for targeted interventions in CRC management.

## 5. Conclusions

In summary, this study highlights a mechanism by which metformin regulates signalling pathways, including PI3K-Akt and MAPK/ERK, through direct miRNA inhibition of genes encoding pathway components and effectors. In particular, metformin upregulated miR-2110 and miR-132-3p to directly target *PIK3R3* and, consequently, modulate the PI3K-mTOR pathway and suppress CRC cell proliferation and cell cycle progression. Similarly, *STMN1* was targeted by upregulated miR-222-3p and miR-589-3p, and its knockdown resulted in cell growth suppression and a delayed cell cycle.

These findings provide new insights into the molecular mechanisms of metformin activity that are responsible for reduced proliferation in CRC cells. With the emergence of RNA therapeutics, future studies are required to explore the clinical utility of these findings.

## Data Availability

Processed data are available in Supplementary Files. The raw transcriptome and small RNA sequencing data are available upon request.

## Supplementary Materials

The following supporting information can be downloaded at: https://www.mdpi.com/article/10.3390/cancers16112055/s1, Figure S1: Pairwise correlation between the differentially expressed genes obtained from total RNA sequencing and targeted RNA panel sequencing. Figure S2: THBS1 gene expression changes associated with exogenous overexpression of miR-590-3p were assessed by real-time RT-PCR and compared with NC mimic. Figure S3: Gene expression changes associated with exogenous overexpression of selected miRNA were assessed by real-time RT-PCR and compared with NC mimic. Figure S4: Original Western Blots. Table S1: Differentially expressed miRNAs associated with 2.5 mM metformin treatment of HCT116 cells and compared with control medium. Table S2: Differentially expressed protein-coding genes associated with 2.5 mM metformin treatment of HCT116 cells and compared with control medium. Table S3: Differentially expressed protein-coding genes from the QIAseq Targeted RNA Panel (human apoptosis and cell death pathway finder RHS-002Z) associated with 2.5 mM metformin treatment of HCT116 cells and compared with control medium. Table S4: Predicted and validated miRNA–mRNA targets showing anti-correlation in expression changes associated with 2.5 mM metformin treatment of CRC cells. Table S5: Enriched KEGG pathways overrepresented with metformin treatment of HCT116. Table S6: miRNA–target gene pairs identified within PI3K-Akt and MAPK pathway. Table S7: Supplementary material and methods.
