# Supplementary material for: Restricting Colorectal Cancer Cell Metabolism with Metformin: An Integrated Transcriptomics Study
Source: Cancers (Basel). 2024 May 29;16(11):2055. doi: 10.3390/cancers16112055 (PMC11171104; doi:10.3390/cancers16112055)

Immunoblots used to create Figure 3 B(i)

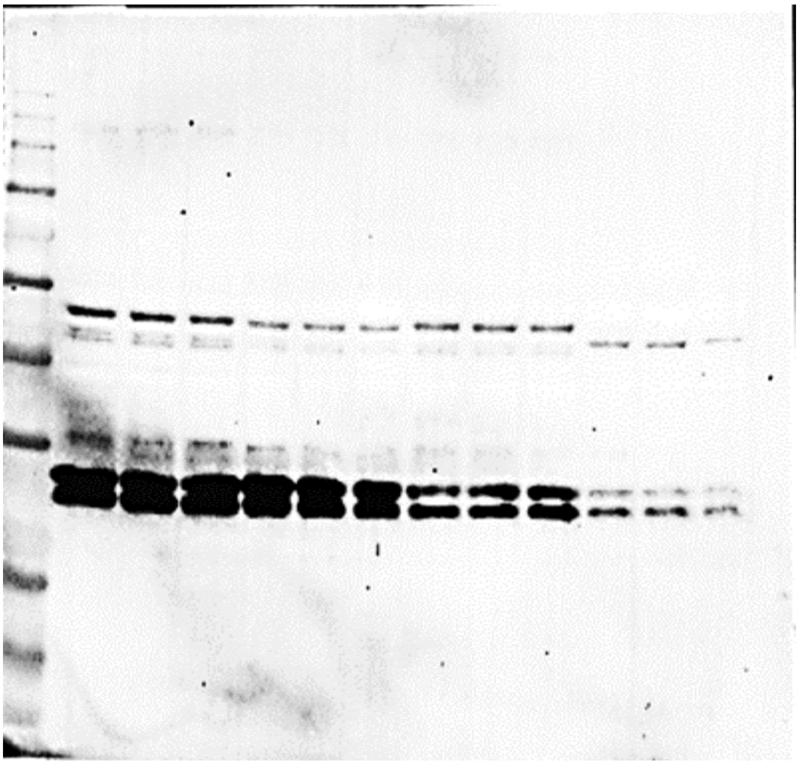

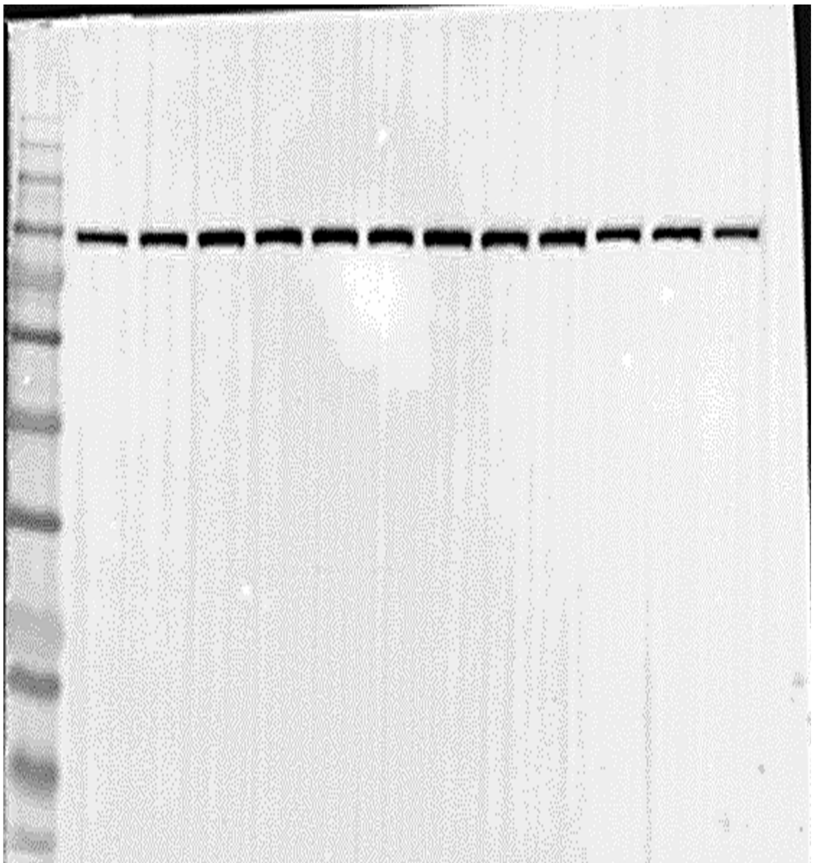

Immunoblots used to create Figure 3 B(ii)

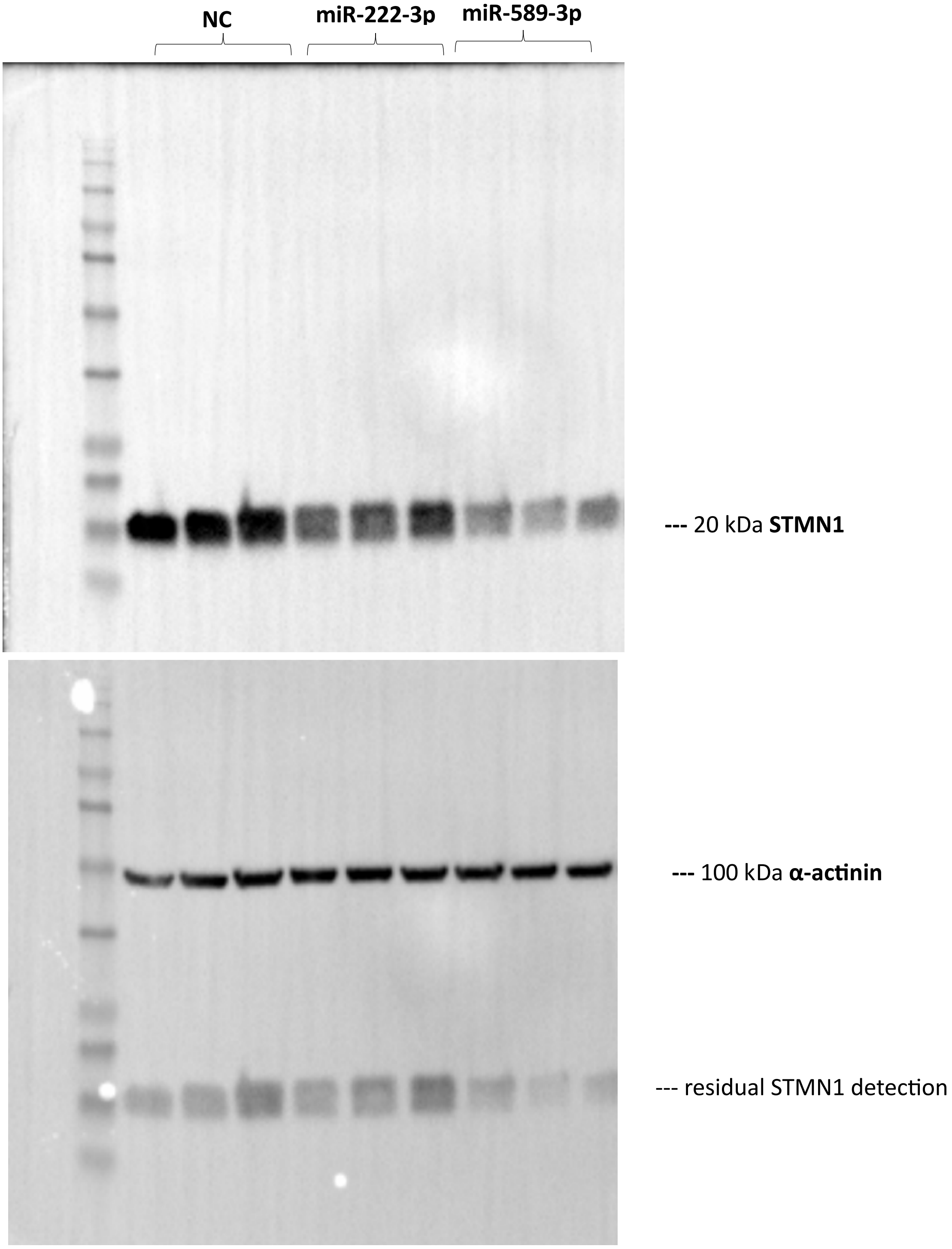

Immunoblots used to create Figure 3 B(ii)

Note: lanes from this blot were selected to create the figure

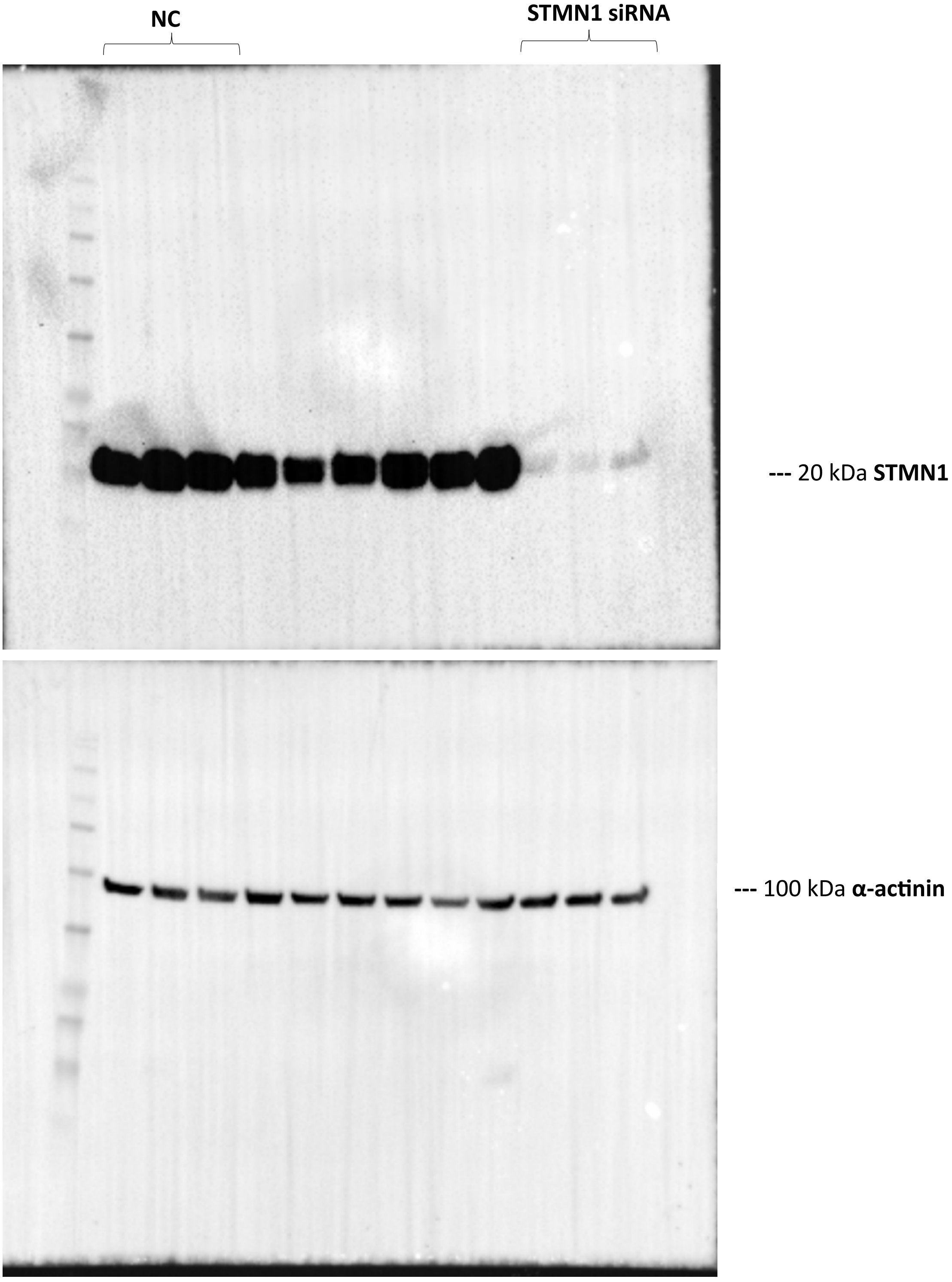

Immunoblots used to create Figure 4 D

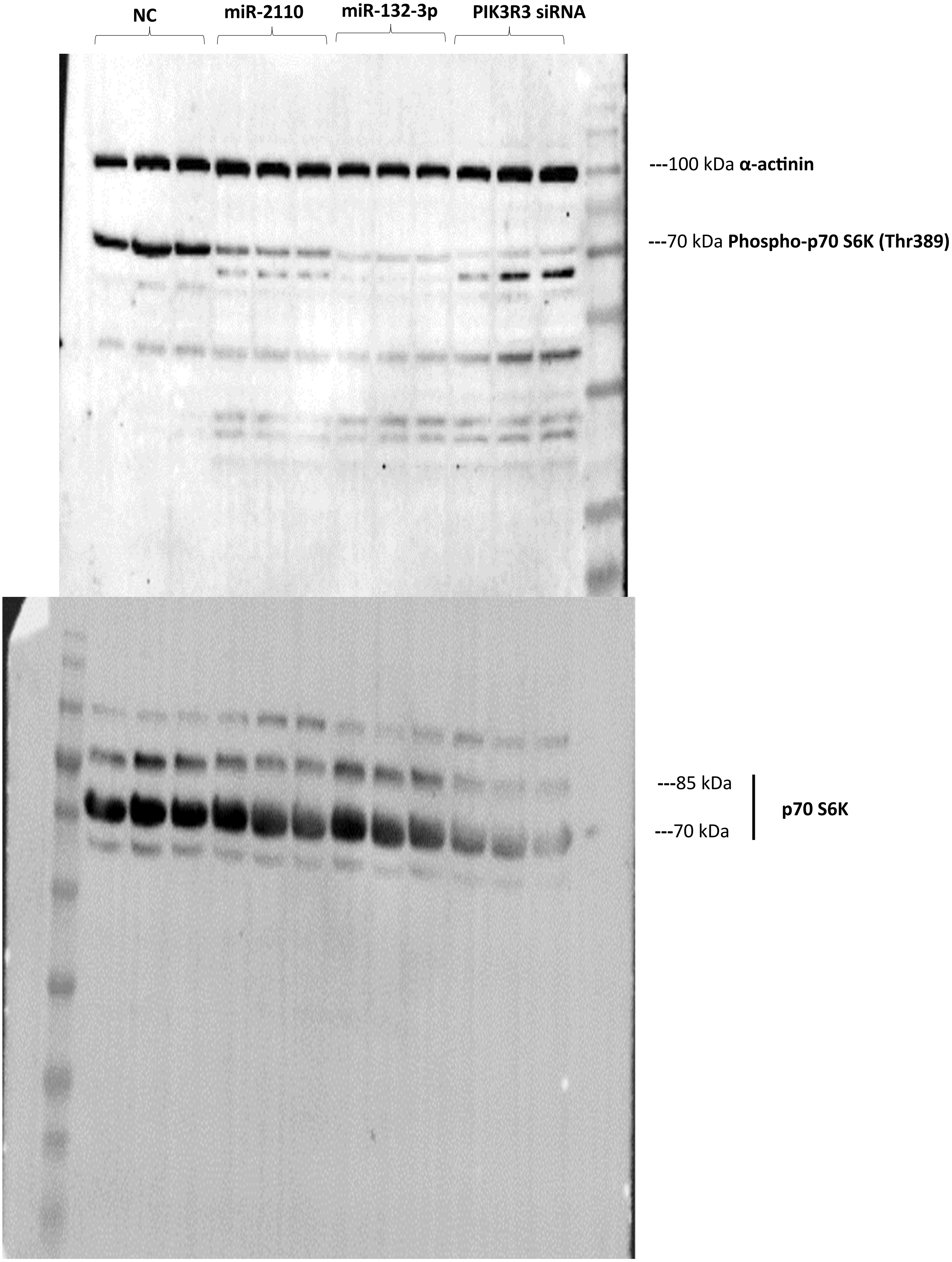

Supplement: Supplementary file 1 [file cancers-16-02055-s001.zip › Supp Figure S4 - Full Immunoblots.pdf]
