# Supplementary material for: Restricting Colorectal Cancer Cell Metabolism with Metformin: An Integrated Transcriptomics Study
Source: Cancers (Basel). 2024 May 29;16(11):2055. doi: 10.3390/cancers16112055 (PMC11171104; doi:10.3390/cancers16112055)
Supplement: Supplementary file 1 [file cancers-16-02055-s001.zip › Supplementary Figures Legends.pdf]

Supplementary Figure S1: Pairwise correlation between the differentially expressed genes obtained from total RNA sequencing and Targeted RNA panel sequencing. Differentially expressed genes associated with 2.5 mM metformin of HCT116 cells were assessed by Illumina total RNA sequencing or QIAseq Targeted RNA Panel. Spearman rank correlation coefficients are shown.

Supplementary Figure S2: (A) *THBS1* Gene expression changes associated with exogenous overexpression of miR-590-3p was assessed by real-time RT-PCR and compared with NC mimic. **(B)** Proliferation of HCT116 cells 96 hours post-transfection with miR-590-3p is shown by quantitative results of crystal violet staining. Results are expressed as mean ± SD of at least 3 replicates and expression levels were normalised to *ACTB* levels. Statistical significance is indicated with asterisk (ns $P > 0.05$, ** $P \leq 0.01$).

Supplementary Figure S3: Gene expression changes associated with exogenous overexpression of selected miRNA were assessed by real-time RT-PCR and compared with NC mimic. Results are expressed as mean ± SD of at least 3 replicates and expression levels were normalised to *ACTB* levels. Statistical significance is indicated with an asterisk (ns $P > 0.05$).

Supplementary Figure S4: Immunoblots used to create manuscript figures are presented in full and labelled accordingly.
