# Supplementary figures and images for: Restricting Colorectal Cancer Cell Metabolism with Metformin: An Integrated Transcriptomics Study
Source: Cancers (Basel). 2024 May 29;16(11):2055. doi: 10.3390/cancers16112055 (PMC11171104; doi:10.3390/cancers16112055)

## Correlation Coefficient

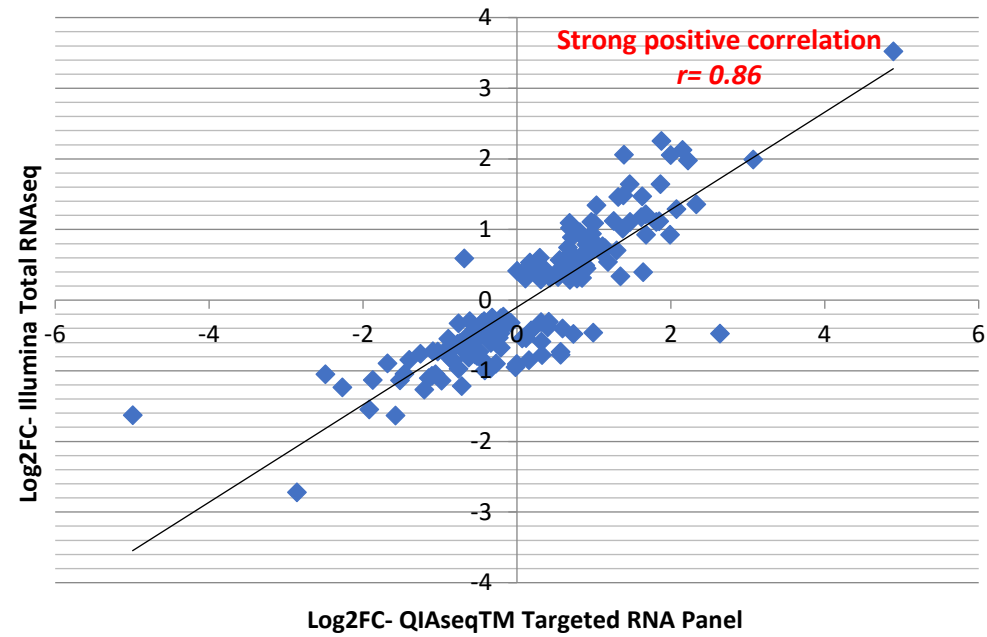

Supplement: Supplementary file 1 [file cancers-16-02055-s001.zip › Supp Figure S1.pdf]

A)

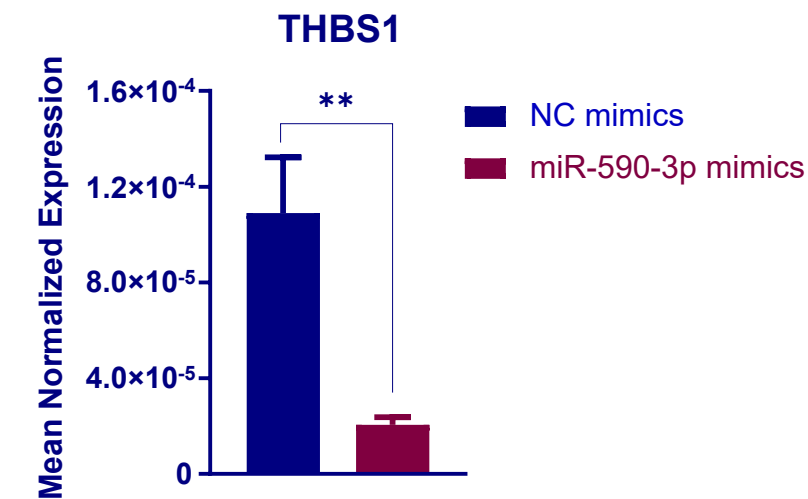

B)

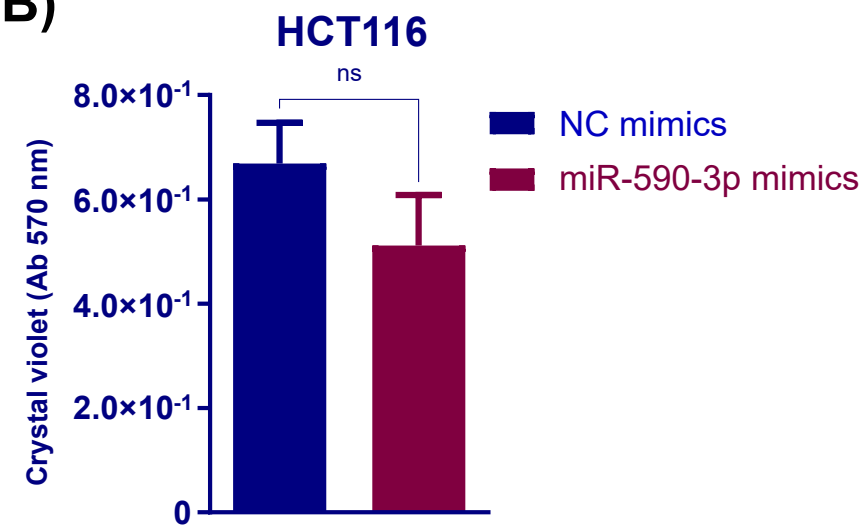

Supplement: Supplementary file 1 [file cancers-16-02055-s001.zip › Supp Figure S2.pdf]

**CDKN1A**

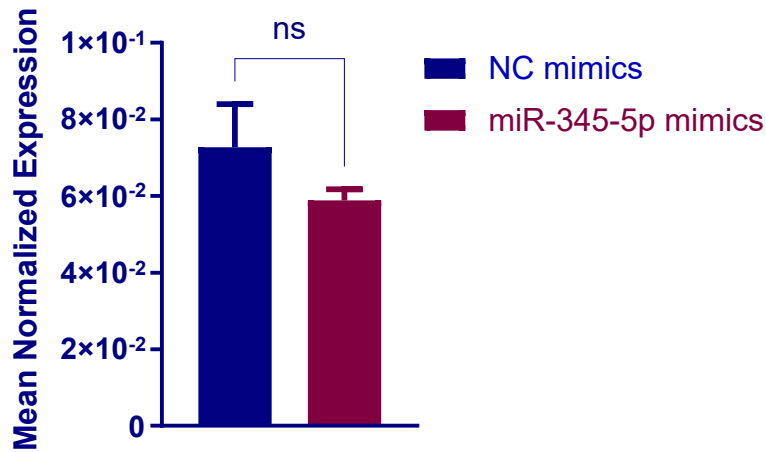

**EFNA4**

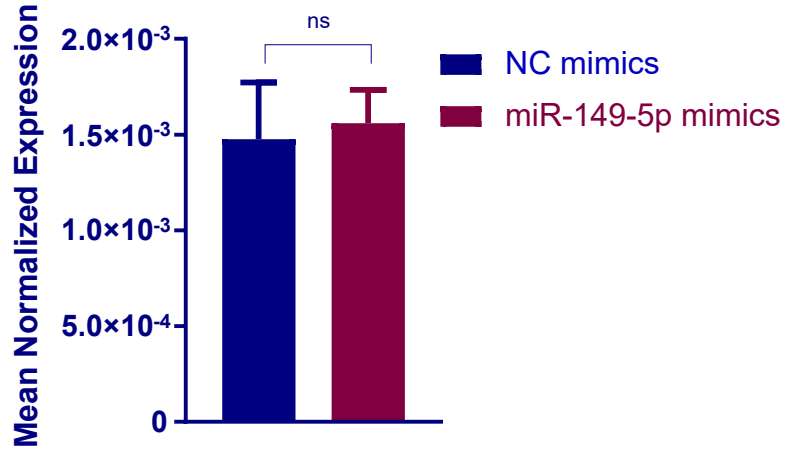

**GADD45A**

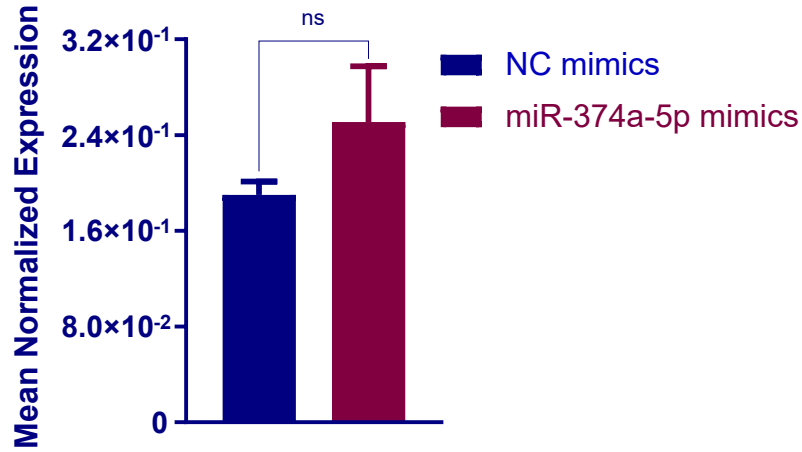

**MYB**

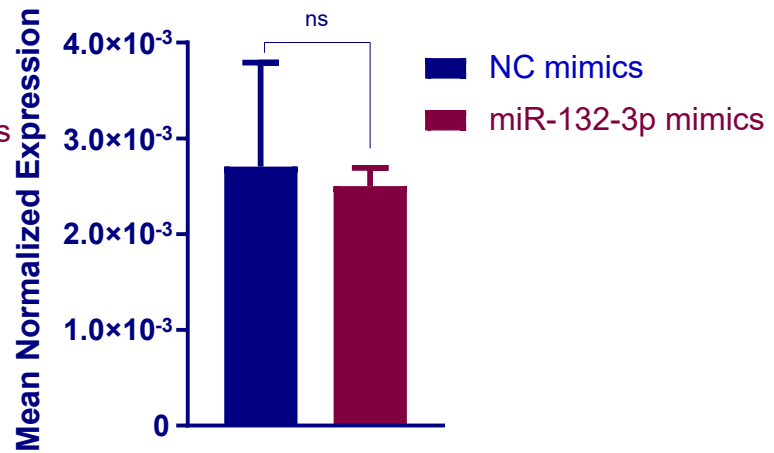

**MECOM**

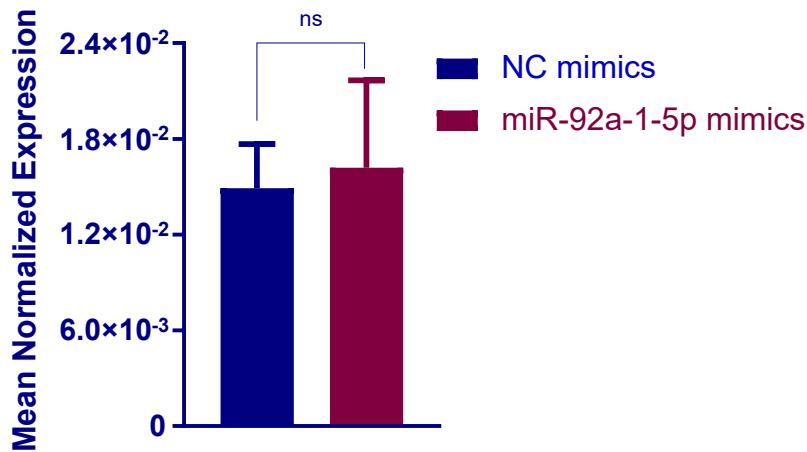

Supplement: Supplementary file 1 [file cancers-16-02055-s001.zip › Supp Figure S3.pdf]
